# Molecular Diagnosis to IgE-mediated Wheat Allergy and Wheat-Dependent Exercise-Induced Anaphylaxis

**DOI:** 10.1007/s12016-025-09059-w

**Published:** 2025-05-05

**Authors:** Witchaya Srisuwatchari, Kantima Kanchanapoomi, Punchama Pacharn

**Affiliations:** Division of Allergy and Immunology, Department of Pediatrics, Faculty of Medicine Siriraj Hospital, Mahidol University, 2 Wanglang Road, Bangkoknoi, 10700 Bangkok Thailand

**Keywords:** Wheat allergy, Anaphylaxis, Diagnosis, Component-resolved diagnosis

## Abstract

IgE-mediated wheat allergy is an emerging problem worldwide, particularly prevalent in Northern Europe and parts of Asia. Another unique manifestation of wheat allergy, wheat-dependent exercise-induced anaphylaxis (WDEIA), and hydrolyzed wheat protein-induced urticaria/anaphylaxis/WDEIA, has increasingly been reported in recent decades. Major wheat protein allergens are classified into two main categories: water/salt-soluble proteins (e.g., alpha-amylase inhibitors, lipid transfer proteins (LTP), and avenin-like proteins) and alcohol/diluted acid-soluble proteins (e.g., gliadins and glutenins). The most allergenic wheat proteins responsible for IgE-mediated wheat allergy are gliadins, particularly omega (ω)-5-gliadin, and glutenins. In cases of WDEIA, ω-5-gliadin and LTP have been identified as the major allergens involved. Diagnostic challenges for IgE-mediated wheat allergy and WDEIA exist due to the variable sensitivity and specificity of currently available tests, including skin prick tests (SPT) and serum-specific IgE (sIgE), which may lead to misdiagnosis. These variations in diagnostic value may be attributed to factors such as clinical presentation, the specific allergens involved, the type of SPT extracts used, and the component tested. Additionally, in countries where grass pollen is a primary sensitizer, in vivo or in vitro cross-reactivity between timothy grass and wheat is common. However, this cross-reactivity is usually asymptomatic and lacks clinical significance. Diagnostic methods have been developed to minimize the risks associated with oral food challenge tests (OFC). Novel approaches, including component-resolved diagnostics (CRD), basophil activation tests (BAT), and epitope-specific antibody assays, provide more precise diagnostic options for IgE-mediated wheat allergy, WDEIA, and its subtypes by targeting specific allergens and components. However, further large-scale studies and validations are required to standardize these diagnostic protocols.

## Introduction

The prevalence of food allergies has increased in recent years, with wheat (*Triticum* spp.) gaining recognition as a significant allergen, alongside cow’s milk, egg, soy, and peanut. Overall, wheat allergy is quite uncommon [1], but in certain regions, such as parts of Asia (including Japan and Thailand), wheat has become a leading cause of food allergies [2–4].

**Fig. 1:**
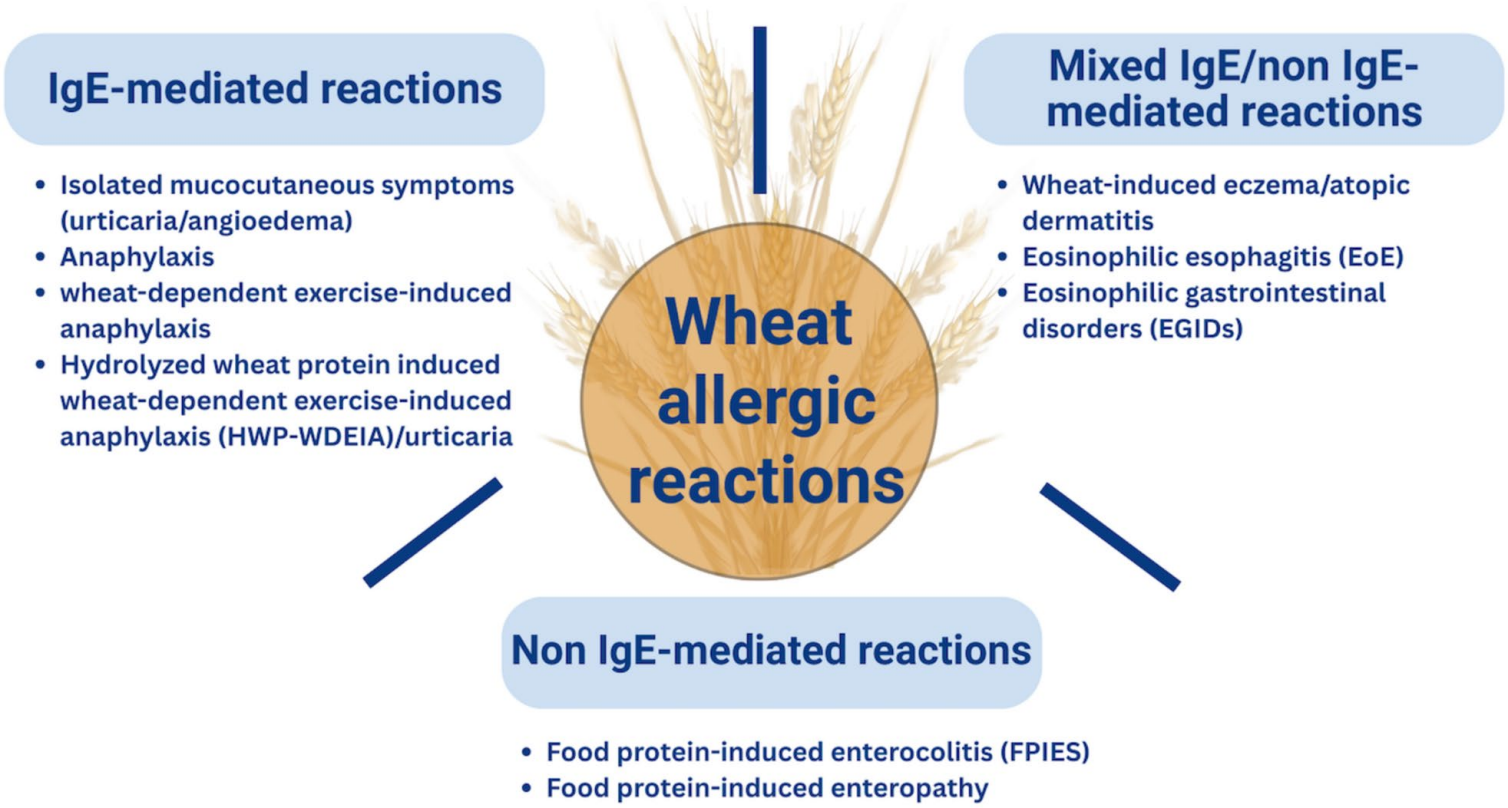
Clinical manifestation of allergic reactions from wheat ingestion

Gluten is the main structural protein of wheat. Adverse reactions to gluten ingestion can be classified into three main mechanisms. These mechanisms include (1) autoimmune (e.g., celiac disease), (2) non-autoimmune and non-allergic (e.g., gluten sensitivity or non-celiac gluten sensitivity, NCGS), and (3) allergic causes. The allergic causes may be from IgE-mediated, mixed IgE/non-IgE-mediated, and non-IgE-mediated reactions (Fig. 1). In IgE-mediated reactions, the clinical presentation can vary from mild mucocutaneous manifestations, such as urticaria and/or angioedema, to life-threatening anaphylaxis. Although T cells are central to the pathophysiology of atopic dermatitis (AD) and eosinophilic gastrointestinal disorders (EGIDs), a significant subset of patients also exhibits concurrent IgE-mediated mechanisms, so they are classified as mixed IgE/non-IgE-mediated reactions. Non-IgE-mediated reactions primarily affect the gastrointestinal tract, manifesting as food protein-induced enterocolitis syndrome (FPIES) or food protein-induced enteropathy [5–7].

Additionally, the IgE-mediated mechanism includes a unique condition known as wheat-dependent exercise-induced anaphylaxis (WDEIA) [8–10]. It is a distinct clinical entity in which anaphylaxis occurs only when wheat ingestion is followed by physical exertion. Recent studies indicate that other cofactors, such as nonsteroidal anti-inflammatory drugs (NSAIDs) or alcohol, may also exacerbate reactions—and, in some cases, trigger symptoms in the absence of exercise. This broader presentation has been referred to as wheat allergy dependent on augmentation factors (WANDA) [11, 12]. Various mechanisms have been proposed to explain how exercise acts as a cofactor in WDEIA; however, supporting data remains limited, highlighting the need for further research to elucidate the underlying pathophysiology [13]. Notably, an outbreak of allergic contact urticaria, anaphylaxis, and/or WDEIA in Japan is linked to the use of facial soaps containing a specific type of hydrolyzed wheat protein [14].

The diagnosis of IgE-mediated wheat allergy typically involves a thorough clinical history, along with skin prick tests (SPT) and/or serum-specific IgE (sIgE) measurements. Wheat component diagnostics, such as sIgE to omega-5 gliadin (ω−5 gliadin), are required in certain cases. Despite combining these methods, the diagnostic accuracy for wheat allergy remains lower compared to other common food allergens. As a result, accurate diagnosis is challenging and often necessitates an oral food challenge (OFC), which is time-consuming and carries significant risks, especially in young children. A study in Thai children reported that 94% of wheat-allergic patients had symptom onset before 1 year of age, and half presented with anaphylaxis [15]. To mitigate risks in very young children, additional assays, such as the basophil activation test (BAT), mast cell activation test (MAT), and epitope-specific B cell antibodies, may aid in diagnosis. While these assays have been explored for diagnosing allergies from peanuts, cow’s milk, and eggs, experimental data on their use for diagnosing wheat allergy remains very limited [7, 16, 17].

**Fig. 2:**
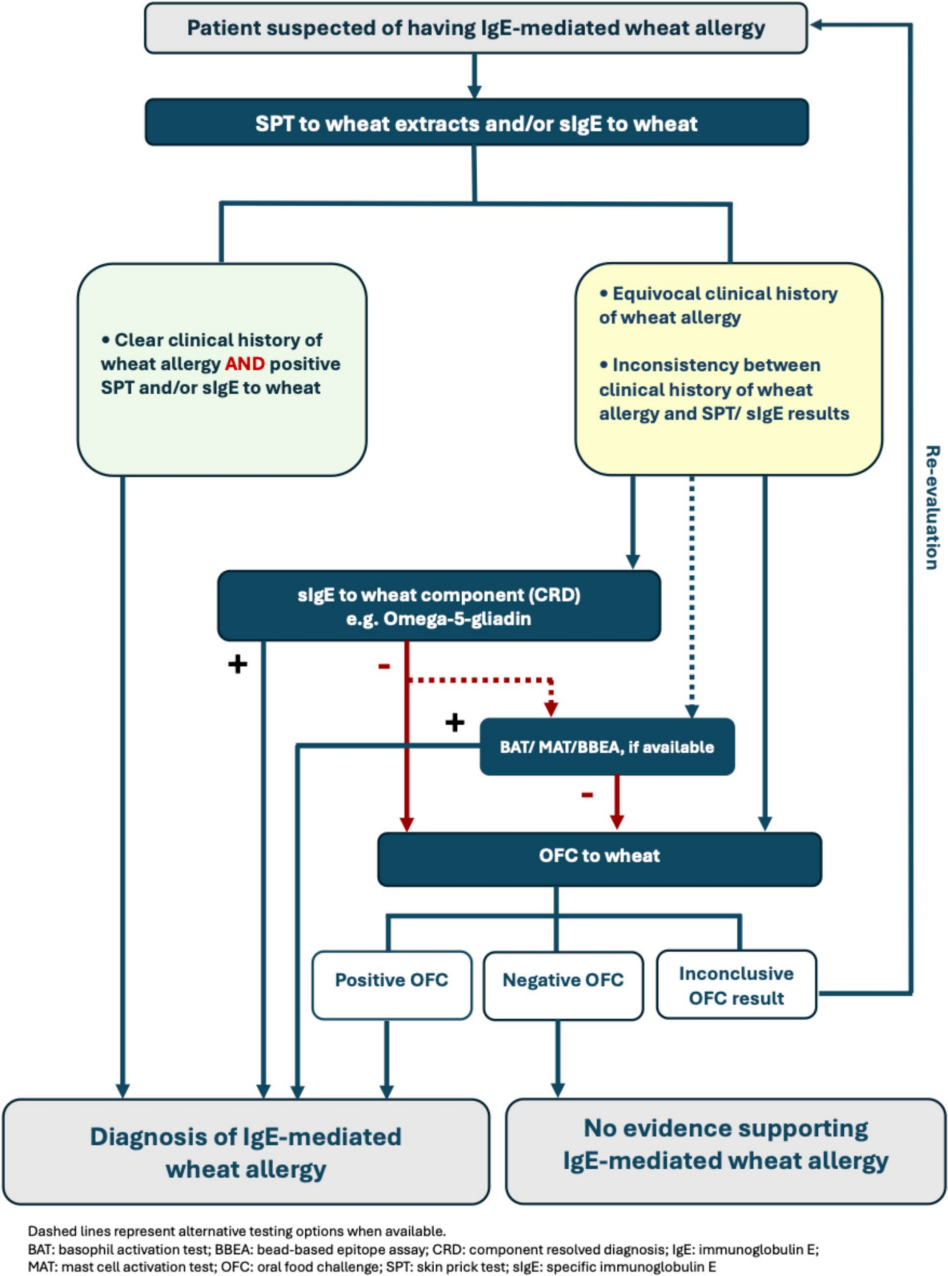
The diagnostic algorithm for IgE-mediated wheat allergy

According to the current recommendations for diagnosing IgE-mediated food allergy by the European Academy of Allergy and Clinical Immunology (EAACI), SPT and sIgE are strongly recommended as first-line diagnostic tests. Component diagnostics are suggested to complement these tests, including sIgE to Ara h 2 for peanut, Cor a 14 for hazelnut, or Ana o 3 for cashew nut. Additionally, BAT for peanut or sesame can be used when available to support the diagnosis in equivocal cases. Furthermore, due to their lack of diagnostic value, the guideline recommends against the isolated use of IgG and IgG subclass tests for diagnosing IgE-mediated food allergy. Finally, a supervised oral food challenge (OFC) is recommended as the definitive procedure to confirm or exclude food allergy [7]. Despite comprehensive reviews of diagnostic tests for food allergies, the literature on wheat allergy remains limited. Therefore, this review will explore the diagnosis of IgE-mediated wheat allergy and WDEIA through laboratory assays targeting relevant molecular wheat proteins and their components, and will propose a diagnostic algorithm to guide clinical practice (Fig. 2).

## Major Allergens of Wheat Proteins

**Fig. 3:**
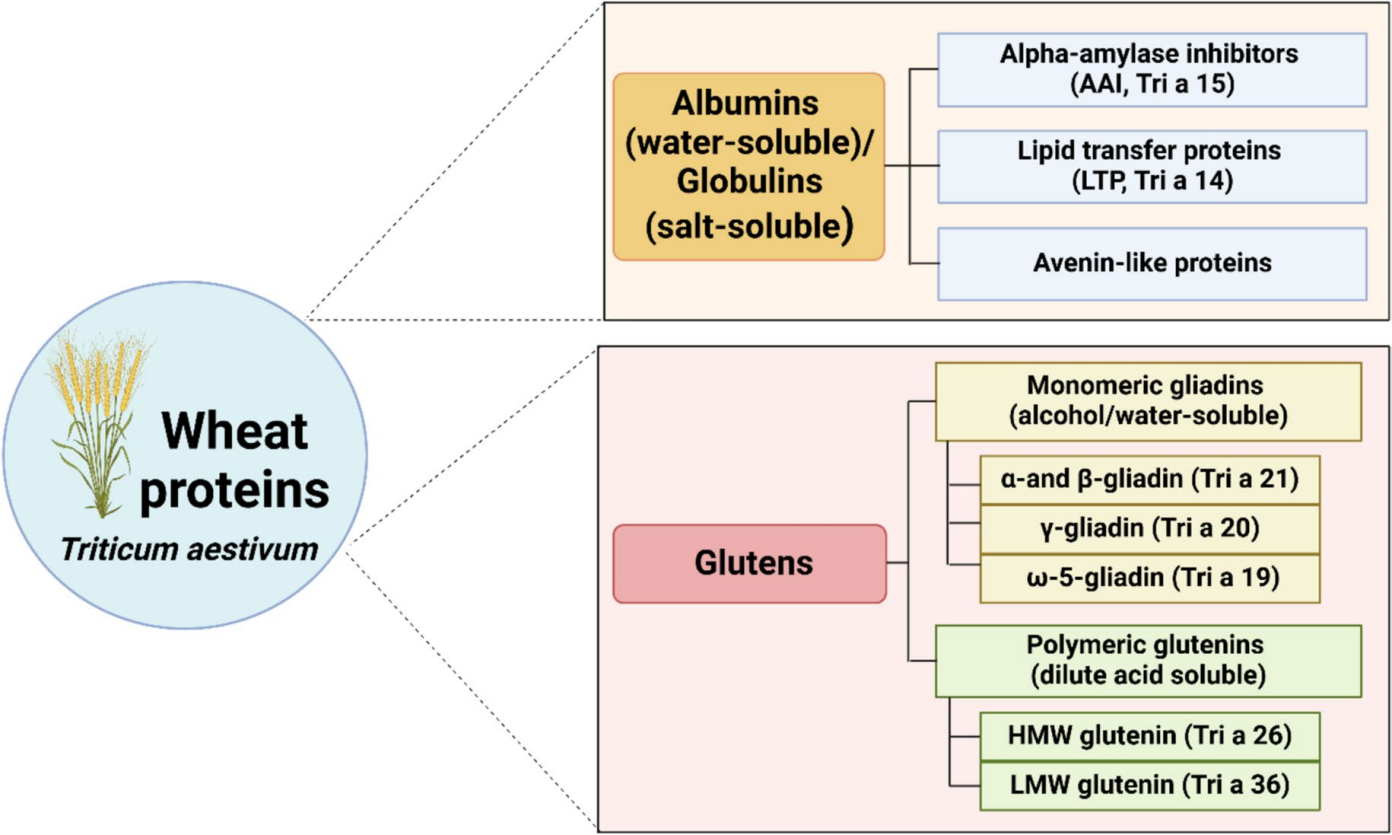
Major wheat protein allergens. Created by BioRender.com/Mahidol University (accessed on January 31, 2025)

Wheat proteins can be classified into two main categories (Fig. 3): water-soluble (albumins)/salt-soluble (globulins) and alcohol/water-soluble (monomeric gliadins) and dilute acid-soluble (polymeric glutenins). The water-/salt-soluble group includes Alpha-amylase inhibitors (AAI, Tri a 15), Lipid transfer proteins (LTP, Tri a 14), and Avenin-like proteins. Gliadins, which are alcohol/water-soluble, can be further subdivided into Alpha (α)/Beta (β)-gliadin (Tri a 21), Gamma (γ)-gliadin (Tri a 20), and omega (ω)−5-gliadin (Tri a 19). Glutenins, which are dilute acid-soluble, consist of High Molecular Weight (HMW, Tri a 26) glutenin and Low Molecular Weight (LMW, Tri a 36) glutenin [18–20]. The most allergenic wheat proteins responsible for IgE-mediated wheat allergy are gliadins, particularly ω−5-gliadin, and glutenins. In cases of WDEIA, ω−5-gliadin and LTP have been identified as the major allergens involved.

Gliadins and glutenins, the major storage proteins in wheat grains, belong to the cereal prolamins family (gluten). Wheat prolamins share a significant degree of sequence and structural homology with their counterparts in barley (hordeins), rye (secalins), oats (avenins), Job’s tears (coixins), and, to a lesser extent, certain types of grasses. This homology leads to extensive in vitro cross-reactivity among these cereal grains. However, clinical cross-reactivity is generally of lesser concern [21, 22].

In countries where grass pollen is the leading cause of sensitization in allergic patients, cross-reactivity between timothy grass and wheat is common. This cross-reactivity involves two known allergens present in both wheat and grass pollen: carbohydrate determinants (CCDs) and profilins. Patients typically have low levels of wheat-sIgE and remain asymptomatic [23]. In a study by Nilsson et al. [23], 43 out of 72 children (59.7%) with a doctor’s diagnosis of grass pollen allergy were sensitized to wheat, with a median sIgE level of 0.5 kUA/L. In contrast, in countries where grass pollen is less prevalent, only 14.8% of 100 children with confirmed IgE-mediated wheat allergy were sensitized to Bermuda or Johnson grass [15]. However, according to a systematic review and meta-analysis by Riggioni et al. [24], the diagnostic accuracy of wheat-sIgE for diagnosing wheat allergy varied by geographical region. When stratified by region, the specificity of wheat-sIgE was lower in Asia (73%) compared to Northern Europe (87%).

## In Vivo and In Vitro Test for the Diagnosis of IgE-mediated Wheat Allergy and WDEIA

### Skin Prick Test (SPT)

SPT is a simple and cost-effective method for diagnosing IgE-mediated wheat allergy with various wheat extracts widely commercially available. However, the effectiveness varies significantly depending on the type of extract used, which critically affects sensitivity and specificity. A study using glycerinated extract reported high sensitivity (81%) but low specificity (64%) [25] (Table 1). Expanding on this, another study compared the diagnostic capacity of an in-house Coca-10% EtOH extract with a commercial extract, demonstrating the superior accuracy of the in-house extract [26]. The increased accuracy likely results from the solubility of gliadin, the major wheat allergen, in alcohol, which enhances the diagnostic value of alcohol-based extracts. Due to the limitations of current diagnostic methods, gliadin and glutenin extracts were developed to improve diagnostic accuracy in wheat allergy. A study among 31 children with wheat allergy found that the gliadin extract with the optimal cut-off point of 2.5 mm demonstrated the highest diagnostic performance, with a sensitivity of 84.2%, specificity of 88.9%, and accuracy of 85.7%. In contrast to the gliadin extract, the commercial extract exhibited significantly lower sensitivity (55%) and accuracy (65.5%). Furthermore, the gliadin extract had the lowest negative likelihood ratio (-LR) among all extracts tested, indicating that a negative gliadin SPT result is highly reliable for ruling out wheat allergy. The stability of the gliadin extract was investigated using SPT and a cell degranulation assay measuring beta-hexosaminidase release [27]. The study found that the extract remained stable for up to 12 months when stored at 2–8 °C, supporting its practicality for long-term clinical use. In a systematic review and meta-analysis by Riggioni et al. [24] and the most recent AAAAI–EAACI PRACTALL guideline, Standardizing Oral Food Challenges—2024 Update [28], a commercial extract of wheat with a cut-off point of 3 mm (IQR 3–5) showed a sensitivity of 53% and a specificity of 72% for the diagnosis of IgE-mediated wheat allergy.

**Table 1:** The diagnostic capacity of skin prick test for IgE-mediated wheat allergy

Study, country	Study population (*n*)	Extract (cut-off, mm)	Sensitivity (%)	Specificity (%)	PPV (%)	NPV (%)	Accuracy (%)	Positive LR	NegativeLR
Eigenmann et al., 1998, USA [25]	21 WA vs. 22 WT	*Glycerinated (3 mm)	81	64	68	78	NA	NA	NA
Palosuo et al., 2001, Finland [35]	19 WA (immediate reactions) vs. 8 WA (delayed reactions) and 13 WT	Wheat in 0.9% NaCl, 1:10 (3 mm)	89	71	74	88	NA	NA	NA
Makela et al., 2014, Finland [18]	30 WA vs. 51 WT	Wheat in 0.9% NaCl,1:10 (3 mm)	NA	NA	56	97	NA	2.14	0.06
		**Gliadin (3 mm)	NA	NA	60	85	NA	2.55	0.29
Pacharn et al., 2020, Thailand [26]	24 WA vs. 6 WT	***Commercial (3 mm)	70.8	100	NA	NA	76.6	NA	NA
		Coca-10% EtOH (3 mm)	91.7	66.7	NA	NA	86.7	NA	NA
Phisitbuntoon et al., 2023, Thailand [37]	22 WA vs. 9 WT	***Commercial (2.5 mm)	55	88.9	91.7	47.1	65.5	4.96	0.51
		Coca-10% EtOH (4 mm)	78.9	77.8	88.2	63.6	78.6	3.55	0.27
		Gliadin (2.5 mm)	84.2	88.9	94.1	72.7	85.7	7.59	0.18
		Glutenin (2.5 mm)	78.9	88.9	93.8	66.7	82.1	7.11	0.24
Riggioni et al., 2024 [24]/Sampson et al., 2024 [28]	388^∔^	Commercial (3 mm)	53	72	NA	NA	NA	NA	NA

Abbreviations: LR, likelihood ratio; mm, millimeter; NA, not applicable; NPV, negative predictive value; PPV, positive predictive value; WA, wheat allergy; WT, wheat tolerant

^*^Glycerinated food extract (1:20), Greer Laboratories, Lenoir, NC

^**^Gliadin 1 mg/ml, Sigma, St. Louis, MO, USA

^***^ALK-Abelló A/S, Hørsholm, Denmark

^∔^From systematic review and meta-analysis

In WDEIA, few studies have evaluated the diagnostic accuracy of the SPT. One study compared the diagnostic performance of SPT using a commercial wheat extract with prick-to-prick (PTP) testing with wheat flour and gluten. All patients with WDEIA tested positive for PTP with gluten, while only 8 of 16 patients tested positive with the commercial extract in the SPT [29], demonstrating the superior performance of PTP testing. In addition, another study using the in-house Coca-10% EtOH extract for diagnosing WDEIA found that 12 of 14 patients tested positive in the SPT [10], indicating variability in the effectiveness of different extracts. This variability is attributed to the solubility of ω−5-gliadin and HMW glutenin, major allergens of WDEIA [30, 31] in alcohol, which explains why the alcohol-based extract showed higher sensitivity than the commercial wheat extract.

Overall, the lower sensitivity of commercial wheat extract for diagnosing IgE-mediated wheat allergy and WDEIA results from the water-insolubility of gliadin, requiring alcohol for proper dissolution.

### Food-Specific IgE (sIgE)

Food-sIgE assays are widely available in clinical settings and are commonly used to diagnose wheat allergy, and they may help predict the severity of the reactions [32]. However, this must be interpreted with clinical history, as a positive result in the absence of clinical symptoms indicates sensitization without clinical significance. The ImmunoCAP assay, a fluorescent method, is currently standard for sIgE quantification. The assay’s various cut-off points offer different diagnostic capacities (Tables 2 and 3). For instance, a cut-off at 26 kUA/L provides 61% sensitivity, 92% specificity, and a negative predictive value (NPV) of 87% [33]. In comparison, a 0.35 kUA/L cut-off showed 96% sensitivity, 20% specificity, and an NPV of 97% [34]. Moreover, a study on Thai children with wheat anaphylaxis found that a 0.35 kUA/L cut-off achieved 100% sensitivity, 50% specificity, and 90% accuracy [26].

**Table 2:** The diagnostic capacity of sIgE for IgE-mediated wheat allergy

Study, country	Study population (*n*)	ImmunoCAP (cut-off, kUA/L)	Sensitivity (%)	Specificity (%)	PPV (%)	NPV (%)	Accuracy (%)	Positive LR	Negative LR
Sampson et al. 1997, USA [34]	23 WA vs. 173 WT	Wheat (0.35)	96	20	14	97	NA	NA	NA
Sampson et al. 2001, USA [33]	23 WA vs. 77 WT	Wheat (26)	61	92	74	87	NA	NA	NA
Palosuo et al. 2001, Finland [35]	19 WA (immediate reactions) vs. 8 WA (delayed reactions) and 13 WT	Wheat (0.35)	95	67	72	93	NA	NA	NA
Ebisawa et al. 2012, Japan [36]	173 WA vs. 138 WT	Wheat (10.1)	61	74	75	60	NA	2.4	0.5
		ω − 5-gliadin (0.41)	72	79	81	69	NA	3.4	0.5
Makela et al. 2014, UK [18]	30 WA vs. 51 WT	Wheat (0.35)	NA	NA	58	97	NA	2.35	0.06
		Gluten (0.35)	NA	NA	56	94	NA	2.16	0.12
		Gliadin (0.35)	NA	NA	53	93	NA	1.9	0.13
Nilsson et al. 2015, Sweden [38] Positive decision point (95% specificity)	32 WA vs. 31 WT	Wheat (70)	62	97	95	71	NA	NA	NA
		ω − 5-gliadin (1.3)	44	97	93	62	NA	NA	NA
		Gliadin (6.0)	69	97	96	75	NA	NA	NA
		HMW-glutenin (1.4)	66	97	95	73	NA	NA	NA
		LMW-glutenin (4.0)	56	97	95	68	NA	NA	NA
Nilsson et al. 2015, Sweden [38] Assay cutoff point	32 WA vs. 31 WT	Wheat (0.35)	100	6	52	100	NA	NA	NA
		ω − 5-gliadin (0.35)	62	84	80	68	NA	NA	NA
		Gliadin (0.35)	94	29	58	82	NA	NA	NA
		HMW-glutenin (0.35)	97	42	63	93	NA	NA	NA
		LMW-glutenin (1.5)	81	52	63	73	NA	NA	NA
Pacharn et al. 2020, Thailand [26]	24 WA vs. 6 WT	Wheat (0.35)	100	50	NA	NA	90	NA	NA
		ω − 5-gliadin (0.1)	83.3	83.3	NA	NA	83.3	NA	NA
		ω − 5-gliadin (0.35)	75	83.3	NA	NA	76.7	NA	NA
		Wheat + ω − 5-gliadin	100	33.3	NA	NA	86.7	NA	NA
Phisitbuntoon et al. 2023, Thailand [37]	22 WA vs. 9 WT	Wheat (0.6)	89.5	66.7	85	75	82.1	2.69	0.16
		ω − 5-gliadin (0.2)	73.7	88.9	93.3	61.5	78.6	6.64	0.3
Riggioni et al., 2024 [24]/Sampson et al., 2024, [28]	1285^∔^	Wheat (0.6)	72	79	NA	NA	NA	NA	NA
	347^∔^	ω − 5-gliadin (0.3)	79	78	NA	NA	NA	NA	NA

HMW-glutenin, high molecular weight-glutenin; LMW-glutenin, low molecular weight-glutenin; LR, likelihood ratio; NA, not applicable; NPV, negative predictive value; ω−5-g, omega-5 gliadin; PPV, positive predictive value; WA, wheat allergy; WT, wheat tolerant

^∔^From systematic review and meta-analysis

**Table 3:** The diagnostic capacity of serum specific IgE for wheat-dependent exercise-induced anaphylaxis

Study, country	Study population (*n*)	ImmunoCAP (cut-off, kUA/L)	Sensitivity (%)	Specificity (%)
Matsuo et al. 2008, Japan [41]	50 WDEIA vs. 25 healthy controls and 25 AD	Wheat (0.35)	48	NA
		Gluten (0.35)	56	44
		rω − 5-gliadin (0.35)	80	68
		rω − 5-gliadin (0.89)	78	96
Brockow et al. 2015, Germany [29]	16 challenge-confirmed WDEIA	Wheat (0.35)	81	87
		Gluten (0.35)	100	95
		ω − 5-gliadin (0.35)	100	97

Abbreviations: AD, atopic dermatitis; NA, not applicable; ω−5-g, omega-5 gliadin; rω−5-g, recombinant omega-5 gliadin; WDEIA, wheat dependent, exercise-induced anaphylaxis

Component-resolved diagnosis (CRD) was developed to identify specific wheat allergens, such as ω−5-gliadin, and improve diagnostic accuracy. The fluorescence enzyme immunoassay (FEIA) ImmunoCAP® method identifies a limited range of wheat allergens, including gliadin, ω−5-gliadin (Tri a 19), and the non-specific lipid transfer protein (ns-LTP, Tri a 14). As a major allergen in IgE-mediated wheat allergy [31, 35], ω−5-gliadin-sIgE may provide a more precise diagnosis than wheat-sIgE [26, 36–38]. Diagnostic yield increases when adding ω−5-gliadin-sIgE, LMW- (Tri a 36), or HMW-glutenin (Tria 26) to wheat-sIgE in the tested panel [38]. In a study of 30 children with a history of IgE-mediated wheat allergy, a 0.35 kUA/L cut-off for ω−5-gliadin-sIgE alone yielded a diagnostic accuracy of 76.7%, whereas combining both tests increased the accuracy to 86.7% [26]. Although Makela et al. [18] found no significant difference in diagnostic capacity between wheat-sIgE and ω−5-gliadin-sIgE using ImmunoCAP, specific IgE measurement via microarray assay improved diagnostic yield, particularly when more than five components tested positive. The improved diagnostic yield may be due to the microarray assay including other wheat allergens, such as α-, β-, γ-gliadin, and water-soluble allergens like alpha-amylase inhibitors (AAI). Distinguishing between patients allergic to ω−5-gliadin and those allergic to other allergens remains challenging, so tests that cover multiple allergens may be more sensitive than single allergen analyses. Considering these findings, no definitive components or sIgE threshold reliably predicts positive wheat challenges. Therefore, allergists should confirm wheat allergy through oral challenges, especially when clinical history is unclear.

While high wheat-sIgE can predict wheat allergy severity [39], wheat anaphylaxis has been reported even in patients with low wheat-sIgE [40]. There were no differences in baseline characteristics between patients with high and low wheat-sIgE, except those with low wheat sIgE showed a more dispersed IgE immunoblot pattern than those with high sIgE levels. The likely allergens in patients with high wheat-sIgE levels were α-, β-, and γ-gliadin. In contrast, those with low wheat-sIgE levels were likely allergic to high molecular weight (HMW) glutenin [40].

In a recent systematic review and meta-analysis by Riggioni et al. [24] and the AAAAI–EAACI PRACTALL guideline Standardizing Oral Food Challenges—2024 Update [28], wheat-sIgE (cutpoint: 0.6 kUA/L, IQR 0.35–5.6) and ω−5-gliadin-sIgE (cutpoint: 0.3 kUA/L, IQR 0.1–0.6) demonstrated sensitivities of 72% and 79%, and specificities of 79% and 78%, respectively, for diagnosing IgE-mediated wheat allergy.

Because ω−5-gliadin and HMW glutenin are the major allergens in WDEIA, ω−5-gliadin-sIgE has better diagnostic capacity than wheat-sIgE [41]. A study of 14 patients with WDEIA found that the median ω−5-gliadin-sIgE level was higher than the wheat-sIgE level (3.8 kUA/L vs 1.3 kUA/L) [10]. In addition, a study of 16 patients with WDEIA found that 3 patients had wheat-specific IgE levels below 0.35 kUA/L, indicating that WDEIA diagnosis might be missed if based solely on wheat sIgE. In contrast, all patients had ω−5-gliadin-specific IgE levels above 0.35 kUA/L [29].

A new subtype of WDEIA has been reported in patients with a history of using soap-containing hydrolyzed wheat protein (HWP). HWP is prepared from the water-insoluble part of wheat protein, followed by enzymatic or acidic hydrolysis. When comparing HWP-WDEIA to conventional WDEIA (CO-WDEIA), HWP-WDEIA showed a higher positive rate of sIgE to wheat and gluten. In contrast, CO-WDEIA showed a higher positive rate of ω−5-gliadin-sIgE. This finding suggests that the hydrolysis process eliminated ω−5-gliadin in HWP.

### Basophil Activation Test (BAT) and Mast Cell Activation Test (MAT)

The BAT is a flow cytometry assay that measures activation markers (e.g., CD63 and CD203c) on basophils after stimulation with specific allergens. BAT has demonstrated a high level of specificity but lower sensitivity compared to current SPT and sIgE tests, except for peanut and sesame allergies, where it shows moderate sensitivity. Therefore, current recommendations suggest using BAT in patients with an uncertain diagnosis of IgE-mediated peanut or sesame allergy to support the diagnosis [7, 28]. One major drawback of this assay is that approximately 10% of the population are non-responders, making interpretation impossible in these cases. Additionally, the assay requires fresh blood and should be conducted using validated methods and standardized conditions. Furthermore, the assay has a dose–response curve, and a standardized threshold has not yet been validated [16].

Currently, no large-scale studies have used BAT to diagnose IgE-mediated wheat allergy. However, a few small cohort studies have evaluated the usefulness of BAT in differentiating IgE-mediated wheat allergy or WDEIA from controls. Furthermore, BAT has been assessed in distinguishing WDEIA subtypes, such as CO-WDEIA and HWP-WDEIA (Table 4) [42–48]. Tokuda et al. [48] demonstrated that the CD203c^high^% induced by native ω−5‐gliadin had a higher area under the curve (AUC) based on ROC analysis compared to wheat-sIgE (CAP-FEIA, Phadia, Tokyo, Japan) for distinguishing patients with IgE-mediated wheat allergy from wheat-tolerant patients. With a cut-off point of 14.4%, the CD203c^high^% test showed 85.0% sensitivity, 77.2% specificity, 86.8% positive predictive value (PPV), and 70.8% negative predictive value (NPV). In comparison, wheat-sIgE with a cut-off of 4.1 UA/mL showed comparable sensitivity (81.4%) and PPV (74.5%) but had lower specificity (55.6%) and NPV (65.2%). BAT has been particularly useful in distinguishing WDEIA patients from controls. For instance, a study by Gabler et al. [45] found that measuring the basophil activation marker CD63 after stimulation with ω−5-gliadin and HMW glutenin effectively identified WDEIA patients. BAT also showed potential in distinguishing WDEIA subtypes. Chinuki et al. [43] demonstrated that patients with CO-WDEIA significantly enhanced CD203c expression in response to purified ω−5-gliadin. In contrast, patients with HWP-WDEIA exhibited CD203c activation in response to the hydrolyzed wheat protein component HWP-A (a component found in Japanese HWP soap manufactured by Katayama Chemical, Osaka, Japan) in a concentration-dependent manner.

**Table 4:** Summary of studies using basophil activation assay in diagnosing IgE-mediated wheat allergy and WDEIA

Study, country	Study population (*n*)	Basophil activation assay		Results	Sensitivity	Specificity	AUC
		Wheat component proteins	Type of basophil activation assay				
IgE-mediated wheat allergy							
Tokuda et al. 2008, Japan [48]	32 WA vs. 27 WT	Water/salt soluble fraction; PBS fraction, water/salt insoluble, EtOH fraction, alkali fraction, native ω−5 gliadin, recombinant ω−5 gliadin	Basophil CD203c expression using a commercial kit (Allergenicity Kit, Beckman Coulter)	Native ω−5 gliadin-induced CD203c^high^% cut-off level of 14.4%	85	77	0.890
				Wheat-sIgE CAP-FEIA (Phadia, Uppsala, Sweden): cut-off level of 4.1 UA/mL	81	56	0.730
Wheat-dependent exercise-induced anaphylaxis							
Chinuki et al. 2012, Japan [43]	5 Conventional (CO)- WDEIA vs. 5 HWP-WDEIA	HWP-A (Katayama Chemical, Osaka, Japan) Purified ω−5-gliadin	Basophil CD203c expression using a commercial kit (Allergenicity Kit®; Beckman Coulter, Fullerton, Calif)	HWP-A enhanced CD203c expression in the patients with HWP-WDEIA Purified ω−5-gliadin enhanced CD203c expression in the patients with CO-WDEIA	NA	NA	NA
Gabler AM et al. 2021, Germany [45]	12 challenge-confirmed WDEIA vs.10 controls	ω5‐, ω1,2‐, α‐, and γ‐gliadins HMW‐glutenin, and LMW‐ glutenin	Basophil activation (%CD63 + basophils), Flow CAST (Buehlmann Laboratories AG)	ω−5‐gliadins: cutoff level of %CD63 + basophils = 1.8	100	70	0.908
				HMW‐glutenin: cut-off level of %CD63 + basophils = 3.0	75	100	0.867
Ogino R et al. 2021, Japan [47]	6 of grass pollen-related wheat allergy (GPWA)	PBS-soluble fraction of wheat proteins	An allergen-induced CD203c expression-based BAT for fractionated wheat proteins using an Allergenicity Kit® (Beckman Coulter, Brea, CA, USA)	All the 6 patients with GPWA were sensitized to water-soluble wheat proteins in BAT	NA	NA	NA
Aoki Yet al. 2023 Japan [42]	14 WDEIA vs. 5 controls (excluding 2 non-responder)	alpha/beta gliadin MM1	BAT (Beckman Coulter, Brea, CA, USA) measured anti-CD203c antibodies	BAT score cut-off value of 10.4%	79	80	NA
Faihs et al. 2024, Germany [44]	13 WDEIA vs. 11 healthy controls	Gluten, HMW glutenin amylase/trypsin inhibitors (ATIs), alcohol-free wheat beer, HWPs, rye gluten and secalins	CD63 + basophils (%CD63 + max)	Basophil activation in patients was significantly higher than in controls for all allergen test solutions (*p* = 0.004–*p* < 0.001)	NA	NA	NA
			Active Basophil Histamine-Release Assay (aBHRA)	Only 50% of the patients exhibited a histamine release greater than the cut-off of 10 ng/mL across all allergens	NA	NA	NA
			Passive Basophil Histamine-Release Assay (pBHRA)	Only 2 patients with the highest sIgE levels against ω5-gliadin exhibited a histamine release	NA	NA	NA

Abbreviations: α-gliadin, alpha-gliadin; AUC, area under the curve; BAT, basophil activation test; γ-gliadin, gamma-gliadin; HMW-glutenin, high molecular weight-glutenin; HWP, hydrolyzed wheat protein; LMW-glutenin, low molecular weight-glutenin; NA, not applicable; ω−1,2-g, omega-1,2 gliadin; ω−5-g, omega-5 gliadin; WA, wheat allergy; WDEIA, wheat-dependent exercise-induced anaphylaxis; WT, wheat tolerance

Interestingly, other wheat proteins measured by basophil activation, including peroxidase-1 (35 kDa) and beta-glucosidase (60 kDa), were also reported to be associated with cases of grass pollen-related WDEIA. These patients tested negative for ω−5-gliadin-sIgE but had high levels of sweet vernal grass pollen-sIgE and timothy grass pollen-sIgE compared to those with CO-WDEIA or HWP-WDEIA [47]. Alpha/beta gliadin MM1 is another reported wheat allergen associated with WDEIA, and it has been suggested to include alpha/beta gliadin MM1 in allergen-sIgE tests to improve the sensitivity for diagnosing WDEIA [42].

MAT is an alternative in vitro diagnostic test similar to the BAT, but instead of using whole blood, the MAT uses plasma or serum to sensitize mast cells. In both tests, the expression of activation markers is measured following stimulation with a food allergen. The MAT has a similar specificity to BAT in diagnosing peanut allergy but exhibits lower sensitivity [17, 28]. For patients with wheat allergy, a study by Bodinier et al. [49] used the MAT to investigate different wheat-induced allergic conditions. The study demonstrated that, in patients with wheat-induced atopic eczema/dermatitis, sera from patients induced enhanced degranulation in response to albumins/globulins extract. In contrast, in cases of WDEIA or IgE-mediated wheat allergy, sera primarily showed degranulation with LMW glutenins, while the albumins/globulins fraction and LTP were rarely positive. Interestingly, in this study, ω−5-gliadin did not appear as a major allergen in the degranulation assays.

### Epitope-Specific Antibodies

The Bead-Based Epitope Assay (BBEA) is a Luminex-based, high-throughput assay developed to simultaneously measure the levels of multiple epitope-specific antibodies, including the IgE, IgG, IgA, and IgD isotypes [50]. The analysis of epitope-specific antibody repertoires has provided novel insights into diagnosing clinical allergies to milk, egg, and peanuts. Additionally, it has been used to predict clinical reactivity, differentiate clinical phenotypes, and predict treatment outcomes in patients who have undergone oral immunotherapy [51–58]. BBEA has shown promising results and requires only a small amount of blood sample. However, the assay has several limitations, including its higher cost than SPT and food-sIgE. Additionally, the assay is only commercially available for peanuts in a limited number of centers.

Recently, this novel tool demonstrated its utility in diagnosing and differentiating the clinical phenotypes of children and adolescents with wheat allergy. The study evaluated 122 patients (83 wheat-allergic and 39 wheat-tolerant) using BBEA to measure epitope-specific (es)-IgE, es-IgG4, and es-IgG1 antibodies against 79 wheat peptides. These peptides were commercially synthesized from α-/β-gliadin, γ-gliadin, ω−5-gliadin, HMW glutenin, and LMW glutenin. Machine learning coupled with simulations identified wheat es-IgE, but not es-IgG4 or es-IgG1, as the most informative for diagnosing wheat allergy. Additionally, the level of es-IgE binding intensity correlated with the severity of wheat allergy phenotypes. Wheat anaphylaxis exhibited the highest es-IgE binding intensity compared to isolated cutaneous symptoms and WDEIA patients. Using a combination of four informative epitopes from ω−5-gliadin and γ-gliadin, BBEA achieved the highest AUC of 0.908, with 83.4% sensitivity and 88.4% specificity. In comparison, wheat-sIgE showed an AUC of 0.646, *P* < *0.001)* [59].

### Oral Food Challenge Test

The OFC remains the gold standard for confirming IgE-mediated wheat allergy when other in vivo and in vitro tests yield inconclusive results. There is no standardized protocol regarding dosing steps or total challenge dose. Additionally, the type of challenge food can vary based on cultural dietary habits, with options including bread, pasta, and udon. In 2020, the Adverse Reactions to Foods Committee of the American Academy of Allergy, Asthma & Immunology published updated guidelines for safely conducting an OFC in clinical settings [60]. For wheat challenges, a slice of bread containing 2–4 g of wheat protein can be used, with portion sizes ranging from ¼ to 2 slices, depending on age. Alternatively, pasta containing 3 g of wheat per cup may be used, with portion sizes ranging from ¼ to 1 cup. Dosing options include a 4-dose protocol (1/12, 1/6, 1/4, 1/2 of the total serving) or a 6-dose protocol (1%, 4%, 10%, 20%, 30%, 35% of the total serving), depending on the risk of severe reactions. Doses are typically administered 15–30 min apart. For WDEIA, the exercise–food challenge test is performed when clinical history, SPT, and/or wheat-sIgE, and wheat components-sIgE testing are insufficient for diagnosis. However, a key limitation of the exercise–food challenge test is its variable positivity rate, which ranges from 50 to 100%, depending on the protocol used [10]. Interestingly, the challenge dose required to elicit a positive reaction in WDEIA is typically higher than in standard OFCs for IgE-mediated wheat allergy, with reported doses ranging from 5.2 to 10.4 g of wheat protein. Optionally, cofactors such as aspirin and/or alcohol can be used to enhance the reaction during the exercise–food challenge test or OFC. Notably, anaphylactic responses in WDEIA tend to be more severe than those seen in typical IgE-mediated wheat anaphylaxis. This increased severity is often attributed to the greater involvement of cardiovascular symptoms (e.g., hypotension, syncope, loss of consciousness) along with respiratory symptoms (e.g., chest tightness, wheezing, cough, desaturation) [8–10, 61].

## Conclusion

The diagnostic accuracy of IgE-mediated wheat allergy using SPT and serum-sIgE measurements remains limited. Combining BAT or epitope-specific antibody assays provides a more accurate approach for diagnosing wheat allergy, WDEIA, and its subtypes. These assays are especially useful when standard tests are inconclusive or less informative, potentially helping to avoid unnecessary oral food challenges. However, these tests still lack large-scale validation, standardized protocols, and defined concentrations and dilutions for in vitro assays. Additionally, concerns remain regarding their availability and cost-effectiveness.

## Data Availability

No datasets were generated or analysed during the current study.

## Abbreviations

α-/β-: Alpha/beta-gliadin
AUC: Area under the curve
BAT: Basophil activation test
BBEA: Bead-based epitope assay
es: Epitope-specific antibodies
γ-gliadin: Gamma-gliadin
HMW glutenin: High molecular weight-glutenin
LMW glutenin: Low molecular weight-glutenin
NPV: Negative predictive value
OFC: Oral food challenge
PTP: Prick to prick
PPV: Positive predictive value
ω-5-g: Omega-5 gliadin
sIgE: Specific IgE
SPT: Skin prick tests
WDEIA: Wheat-dependent exercise-induced anaphylaxis
